# Editorial: Opportunities and challenges of interprofessional collaboration and education, volume II

**DOI:** 10.3389/fmed.2025.1598331

**Published:** 2025-04-16

**Authors:** Jill E. Thistlethwaite, John Gilbert, Anthony P. Breitbach, Ciraj Ali Mohammed, Martina Müller-Schilling, Sophie Schlosser-Hupf

**Affiliations:** ^1^Faculty of Health, University of Technology Sydney, Sydney, NSW, Australia; ^2^UBC Emeritus College, University of British Columbia, Vancouver, BC, Canada; ^3^Interprofessional Education Program, Saint Louis University, St. Louis, MO, United States; ^4^Medical Education, National University of Science and Technology, Muscat, Oman; ^5^Department of Internal Medicine I, Gastroenterology, Hepatology, Endocrinology, Rheumatology and Infectious Diseases, University of Regensburg, Regensburg, Bavaria, Germany

**Keywords:** interprofessional, interprofessional education, interprofessional collaboration, teamwork, interprofessional practice, interprofessional learning

This is the second collection of articles for the Research Topic: opportunities and challenges of interprofessional collaboration and education. The 12 papers showcase the diversity of interprofessional approaches to health care challenges influenced by the local context including healthcare and education systems and resources. Five contributions come from Germany due to the strong support for interprofessional education (IPE) through its planned integration into the national medical curriculum. Additionally, an expanding interprofessional network is currently forming. IPE research and initiatives receive funding from organizations such as the Robert Bosch Foundation, further driving its development in Germany.

Many universities and jurisdictions have adopted the language of competency-based education (CBE) for interprofessional education (IPE). Competence may be defined succinctly as what health professional graduates should be able to do in clinical practice (1). In their Perspective, Williams et al., provide guidance for healthcare institutions seeking to implement effective IPE for students. They emphasize the importance of preparation to be “team ready” on graduation. The article acknowledges ongoing challenges in healthcare education but offers practical solutions based on the authors' extensive experiences. The goal is to create sustainable interprofessional curricula that enhance collaboration among future healthcare professionals and minimize negative impacts of professional stereotypes.

Once an interprofessional program has defined its competencies, attention turns to the means of assessing these. There are increasing numbers of tools and instruments developed for such assessment. Brownie et al.'s scoping review focuses on tools used for self- and peer assessment. It describes 20 studies and 12 tools, discussing the strengths and weaknesses of each. The paper is a useful resource for educators but highlights the need for a consensus approach to assessment, particularly to support learning effectively.

To achieve interprofessional competencies for subsequent health professional practice, relevant learning activities are required. These may be in the formal curriculum, or informal and extra-curricular activities developed by the learners themselves. Hirsch et al. at the University of Birmingham (UK) studied the impact of an innovative IPE student society, the Knowledge and Skills Exchange (KASE), on participants' subsequent experience as health professionals. Through interviews, the authors identified positive perceptions around themes of interprofessional communication, teamworking, patient-centered care, leadership and organizational skills, confidence and resilience. These findings align with research that early exposure to IPE, relevant to the context of students' lived experience, can have a positive impact on their professional practice including team collaboration, the quality of care provided and job satisfaction, all of which have been shown to contribute to improved health outcomes (2–6).

Formal learning includes interprofessional training wards (IPTW), i.e. functioning inpatient wards staffed by students working collaboratively under supervision. These wards continue to be evaluated as authentic interprofessional clinical activities. Three papers from Germany focus on IPTWs. Schlosser-Hupf et al. looked at the cost-effectiveness of an internal medicine IPTW, an important evaluation as costing health professional education is difficult and rare (7). The A-STAR IPTW at University Hospital Regensburg was compared to conventional wards. The research analyzed 7,244 patient cases examining economic outcomes and clinical performance. This study demonstrates that IPTWs can be economically viable while providing quality care, even during challenging periods like the COVID-19 pandemic. The authors suggest these findings provide a compelling rationale for broader implementation of such wards as platforms for educating future healthcare professionals. Schwarz et al. report on an IPTW in neonatology at a Munich hospital that had a positive impact on IPL and self-assessment of competencies. Emphasizing the importance of training in interprofessional facilitation, Müller et al. in Freiburg developed and evaluated a faculty development program for medical residents engaging with an IPTW.

IPE is thriving in parts of Africa. The African Interprofessional Education Network (AfriPEN) works of the sub-Saharan region hosted its 4th conference in 2023 on the topic: Are we making a difference in Africa (8)? Helping to answer this question Nawagi et al. present an evaluation of one African interprofessional initiative: the AFREhealth-FAIMER IPECP student elective exchange program. The African Forum for Research and Education in Health (AFREhealth) and the Foundation for the Advancement of International Medical Education and Research (FAIMER) supported this program for 13 institutions in 10 African countries. Students participated in a six-week virtual clinical interprofessional learning activity based on r case studies. While the evaluation findings are short-term, they do indicate the value of this type of cross-country activity contributing to skill development for collaborative practice. The authors advocate for longitudinal studies to examine how IPL translates into behavior change and practice.

IPE for the development of an interprofessional identity is being increasingly recognized globally. Reinders et al. explore the extended professional identity theory (EPIT) as a framework for fostering interprofessional identities that complement individual professional identities. The authors consider how through integrating interprofessional identity formation with skill development and environmental adaptability, EPIT enhances collaboration in diverse professional settings. The discussion highlights EPIT's potential in Türkiye, particularly in advancing IPE, university engagement, and collaborative strategies while addressing local challenges.

IPECP continues to be important for health professionals following qualification. Fleischmann et al. implemented an interprofessional approach to improve medication management for patients with inflammatory bowel disease (IBD). The findings of this prospective study demonstrated that integrating pharmaceutical expertise into IBD care significantly improves patient satisfaction, reduces medication-related concerns, and enhances medication safety. The authors advocate for routine medication reviews to optimize therapeutic outcomes and better integrate patient perspectives into clinical practice.

The outcomes of IPECP are being researched not only in terms of patient care but also effects on health professional wellbeing. Ruttmann et al. examined the relationship between interprofessional collaboration and psychological distress among healthcare professionals during the COVID-19 pandemic at a German university hospital. The monocentric cross-sectional study was conducted during the initial pandemic wave and involved 299 healthcare professionals. It highlights the vital role of enhanced interprofessional collaboration in strengthening healthcare professionals' psychological wellbeing during crises. The authors emphasize the need to foster collaborative environments and integrate IPE to help build resilience in healthcare teams. Professional wellbeing and patient outcomes are also affected by organizational culture. While the survey instrument developed by Rietdijk et al. to assess self-perceived open organizational culture was validated in a hospital pharmacy, it has the potential to be employed in interprofessional practice settings.

The development and use of applications (apps) for health professional learning on mobile devices are becoming more widespread. Seelandt et al. evaluated the impact of an evidence-based debriefing app on anesthesia team performance. The researchers observed anesthesia teams during two complex inductions, with teams using the Zurich Debriefing App between procedures. This small pilot study indicates that the app enhances anesthesia team performance, particularly through senior physicians' reflective contributions. The researchers note that the app offers a resource-efficient way to integrate debriefing into clinical practice, potentially improving interprofessional team functioning and patient safety.

Opportunities and Challenges of Interprofessional Collaboration and Education II presents diverse perspectives from education and practice. It provides evidence that the interprofessional field is growing in maturity and rigor. Researchers must continue to move from studies that are narrowly defined in single experiences to exploring interventions across multiple contexts, over time and situated in authentic experiences. This will provide added insight into how IPECP impacts all learners throughout the continuum of health professional education and practice in addition to patient/client outcomes.
